# Insulin like growth factor-I in acute subarachnoid hemorrhage: a prospective cohort study

**DOI:** 10.1186/cc8988

**Published:** 2010-04-28

**Authors:** Stepani Bendel, Timo Koivisto, Olli-Pekka Ryynänen, Esko Ruokonen, Jarkko Romppanen, Vesa Kiviniemi, Ari Uusaro

**Affiliations:** 1 Department of Intensive Care, Kuopio University Hospital and University of Eastern Finland, PL 1777, 70211 Kuopio, Finland; 2 Department of Neurosurgery, Kuopio University Hospital and University of Eastern Finland, PL 1777, 70211 Kuopio, Finland; 3 Department of Public Health, University of Eastern Finland, Asemakatu 44 A 4, 70100 Kuopio, Finland; 4 Eastern Finland Laboratory Centre, Kuopio University Hospital, PL1777, 70211 Kuopio, Finland; 5 IT Centre, Statistical and mathematical sciences, University of Eastern Finland PL 1627, 70211 Kuopio, Finland

## Abstract

**Introduction:**

Neuroendocrine deficiencies may affect recovery after aneurysmal subarachnoid hemorrhage (aSAH). Insulin like growth factor-I (IGF-I) regulates neuronal growth and apoptosis in ischemic stroke. Our study was designed to a) characterize the behavior of serum IGF-I and growth hormone (GH) in the acute and late phases after aSAH reflecting possible pituitary gland function and b) evaluate the association between IGF-I and morbidity assessed by Glasgow outcome scale (GOS) and health related quality of life (HRQoL) in patients with aSAH.

**Methods:**

In this prospective cohort study, patients with aSAH (n = 30) were compared to patients who underwent elective aneurysm surgery (n = 16). Serum GH and IGF-I concentrations were measured daily for five (controls) or seven (aSAH) days and at three months. GOS and 15d HRQoL was measured at three months. A mixed models method was used for testing between the groups. For factors possibly affecting HRQoL in aSAH patients, we constructed a Bayesian predicting model using a P-course Bayesian classifier.

**Results:**

The mean IGF-I concentrations for days one to five were 8.1 ± 3.5 nmol/l in patients with aSAH and 11.2 ± 3.1 in the control group (*P *= 0.01). No corresponding difference was found at three months. Serum GH concentrations were similar in both patient groups. Severity of the aSAH did not affect serum IGF-I concentrations. Patients with GOS ≤ 4 had lower IGF-I concentrations and lower HRQoL than patients with GOS 5 (*P *= 0.02 and 0.003 respectively). The 15d HRQoL was 0.81 ± 0.16 in patients with aSAH and 0.86 ± 0.09 in control patients (*P *= 0.24). In the Bayesian model, the use of statins prior to aSAH, hyponatremia, high maximal sequential organ specific score (SOFAmax), and low cumulative IGF-I concentrations on days one to seven were associated with poor HRQoL (accuracy 89%, sensitivity 86%, and specificity 93%).

**Conclusions:**

IGF-I concentrations are low during acute aSAH, which may have an impact on morbidity.

**Trial registration:**

ClinicalTrials.gov Identifier NCT00614887

## Introduction

Acute aneurysmal subarachnoid hemorrhage (aSAH) is a devastating disease, annually affecting 9 in 100,000 people [1]. The outcome of aSAH may be affected by major neurological deficits and cardiovascular, endocrinological, or psychological disorders. Neuroendocrine deficiencies may also affect recovery and rehabilitation after aSAH. Endocrinological disturbances may develop because of the proximity of the hypothalamus and hypophysis to sensitive vascular structures, which can be affected by acute aSAH. Hydrocephalus, local hemorrhages, microinfarctions, venous stasis, vasospasm, and surgical manipulation may also cause dysfunction of the pituitary gland and/or hypothalamus [2].

Some studies suggest the hypothalamo-pituitary-adrenal (HPA) axis may already be affected in the acute phase of aSAH [2-6]. Several studies have revealed that, at least in the late phase, aSAH may present with a pituitary insufficiency [2,4,7,8]. Growth hormone (GH) deficiency is the most common single pituitary hormone deficit in patients with traumatic brain injury (TBI) and aSAH [4,8]. In patients with aSAH, GH deficiency may affect the quality of life [9]. GH mediates its action via insulin-like growth factors (IGF-I). IGF-I has potential effects on neuronal growth but also on neuronal cell death, apoptosis and neuromodulation [10-12], which play a major role in ischemic stroke and in the pathological cellular processes of aSAH [13]. Levels of IGF-I are low during the acute phase of critical illness [14]. Recent studies suggest low IGF-I levels may negatively affect outcome, at least in patients with ischemic stroke [15-17], and low IGF-I values in patients with hemorrhagic stroke may be associated with excess mortality [17].

There is no data available on the behavior of serum IGF-I concentrations in the acute phase of aSAH. The aim of our study was to characterize the behavior of IGF-I and GH acutely and three months after aSAH. This may reflect acute pituitary function. Additionally, we hypothesized that low cumulative IGF-I concentrations may negatively influence morbidity assessed by Glasgow outcome scale (GOS) and health-related quality of life (HRQoL).

## Materials and methods

All patients aged 18 years or over and scheduled for an elective open surgical aneurysm treatment (control group), and all patients with aSAH admitted to Kuopio University Hospital in Finland between 29 March and 30 November 30, 2006 were prospectively assessed for eligibility for this study. Patients with an elective endovascular management of their unruptured aneurysm were excluded from the control group because of their expectedly short hospital stay. This study's patient population is the same as that used in a previous publication [3]. The exclusion criteria were: any known pituitary insufficiency, use of etomidate before study entry or during the study period, unknown exact bleeding day, bleeding more than three days before inclusion, previous aSAH, and moribund state of the patient.

The hospital ethics committee approved the study protocol and informed written consent was received from the patients or their next of kin.

### Patients with aSAH

The following blood samples were collected from the first to seventh mornings following bleeding: serum (s) GH (reference value 0 to 11.5 mU/l), serum IGF-I (reference values: 15 to 45 nmol/l for age 21 to 30 years; 14 to 36 nmol/l for age 31 to 50 years; ages 10 to 29 nmol/l for age 51 to 70 years; and 8 to 23 nmol/l for age over 70 years). In control patients, the corresponding blood samples were collected from the first to fifth postoperative days after the patients were discharged. Additional routine laboratory parameters, such as electrolytes, were collected on a daily basis.

### Follow up at three months

At the scheduled three-month follow-up visit, serum GH and serum IGF-I samples were collected at 9 am. In addition, the patients (or their relatives) were asked to fill out a 15D quality of life questionnaire [18]. We used age-matched and sex-matched IGF-I-concentrations as indicators for low IGF-I and suspected GH deficiency. We also used levels of IGF-I less than 11 nmol/l [19] as the cut off for low IGF-I. Samples of serum GH and serum IGF-I were stored at -70°C for analysis. The same personnel performed all analyses in one laboratory at the Kuopio University Hospital. Serum GH concentrations were analyzed with specific time-resolved fluoroimmunoassay by AutoDelfia (PerkinElmer Life and Analytical Sciences Wallac Oy, Turku, Finland). Serum IGF-I concentrations were analyzed using a quantitative sandwich ELISA technique (Quantikine Human IGF-1 Immunoassay; R&D Systems, Minneapolis, MN, USA).

### Quality of life assessment

The HRQoL was measured by the 15D scale [18,20]. The 15D is a generic and standardized HRQoL instrument consisting of 15 dimensions: mobility, vision, hearing, breathing, sleeping, eating, speech, elimination, usual activities, mental functioning, discomfort and symptoms, depression, distress, vitality, and sexual activity. Each dimension has five grades of severity. For each dimension, the respondent must choose one of the five levels that best describes his or her state of health at the moment (best level = 1; worst level = 5). The results of 15D can be presented as a single index or as a profile of all 15 dimensions. A change of 0.02 to 0.03 points in the health utility index or 15D score is considered to be clinically noteworthy. The values on a 0 to 1 scale reflect the levels of the dimension, with 1 corresponding to no problems with the dimension and 0 to being dead. The mean score of the Finnish population aged 50 to 59 years was 0.92 (0.91 to 0.92) [21]. In this study, HRQoL-indexes were classified into three groups: 0.8 to 1.0 = normal, 0.6 to 0.79 = limited, and less than 0.6 = poor HRQoL.

### Statistical methods

We used a power of 80% and a two-sided α-level of 0.05 in sample size calculations. We assumed that 25% of patients with aSAH and none of the elective surgical patients would develop pituitary insufficiency measured by low IGF-I. Data are presented as mean ± standard deviation, absolute values and percentages, or medians and interquartile ranges. Distribution of the data was assessed by the Kolmogorov-Smirnov test. For normally distributed parameters, student's t-tests were used to compare the means of different groups. The Mann-Whitney U test was used for nonparametric testing between the groups. A mixed models method was used to test between groups, allowing heterogeneity between the groups. To identify the factors associated with poor HRQoL or death, we used a Bayesian predicting model in aSAH patients. This was performed using P-course Bayesian classifier [22].

P-course is a web-based Bayesian classifier that is able to use multidimensional priors, for example separate priors for the outcome variable, in general, and for the outcome variable according to each predicting variable. The methods have equaled or outperformed novel logistic regression, especially in small data sets, in terms of prediction accuracy [23], variable selection, and multiple performance measures. They can perform well with incomplete or complex data typical to small data sets. Modeling of this data was made without informative *a priori *information.

The outcome variable was poor HRQoL measured by 15D and dichotomized into normal (0.80 to 1.00) or poor (0 to 0.79), where the value 0 indicated death. In the first phase, there were 355 potential predicting variables. By using P-course classifier, this was reduced to 22 variables from 30 aSAH patients. To avoid over-fitting the model, we formed four randomly selected sets of 25 patients, and a prediction model was performed for each set. We obtained four slightly different sets of prediction variables.

## Results

We recruited 30 patients with aSAH and 16 control patients who underwent elective aneurysm surgery. We were unable to recruit the planned 30 control patients because endovascular aneurysm treatment was frequently chosen [3]. Demographic patient data are presented in Table 1.

**Table 1:** Patient demographics

	aSAH Clipped (n = 13)	aSAH Coiled (n = 17)	*P *value for clipped vs. coiled	Control (n = 16)	*P *value for aSAH vs. controls
Age, years (range)	50 (25-73)	54 (21-78)	0.53	50 (37-64)	0.53
Gender M/F	5/8	9/8	0.41	4/12	0.15
**Aneurysm location**					
ICA	2	4		6	
MCA	8	0		10	
ACoA	1	10		0	
ACA distal	1	1		0	
VBA	1	2		0	
Hydrocephalus at admission	2	4		0	
**Fisher grade**					
I-II	2	2			
III-IV	11	15			
**Hunt and Hess initial**					
I-II	5	10			
III	3	3			
IV-V	5	4			
SAPS II	27 ± 12	33 ± 14	0.27		
APACHE II	14 ± 5	16 ± 6	0.53		

Data are presented as numbers or mean ± standard deviation unless otherwise indicated.

aSAH clipped, patients with their ruptured aneurysm treated by open surgical clipping, aSAH coiled, patients with their ruptured aneurysm treated by endovascular coiling. ACA, anterior cerebral artery; AcoA, anterior communicating artery; APACHE, Acute Physiology and Chronic Health Evaluation; ICA, internal carotid artery; MCA, median cerebral artery; SAH, subarachnoid hemorrhage; SAPS, Simplified Acute Physiology Score; VBA, vertebro basilar artery.

Seventeen patients with aSAH underwent endovascular treatment and 13 patients had open surgery. The mean length of stay (LOS) was 97 ± 100 hours at the ICU and 34 ± 51 hours at the high-dependency unit (HDU) in patients with aSAH. Patients in the control group had an HDU LOS of 22 ± 2 hours. Patients with aSAH had a hospital LOS of 14.6 ± 5.4 days, and patients in the control group of 8 ± 3 days (*P *< 0.001). The three-month mortality was 10% in patients with aSAH. None of the patients in the control group died.

**Figure 1:**
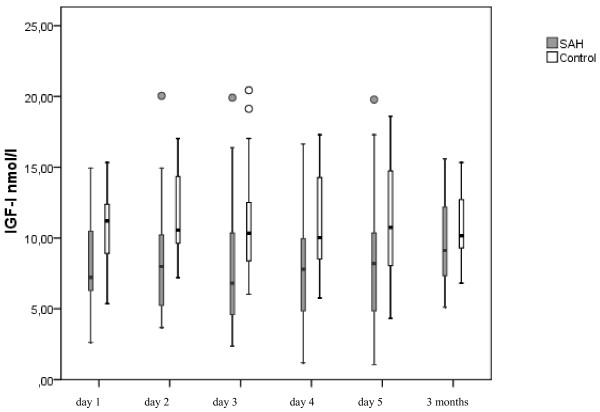
**Serum IGF-I concentrations in patients with aSAH and in the control group**. *P *= 0.01 for the difference between groups on days one to five and *P *= 0.9 at three months. Data are presented as median, interquartile ranges, and outliers. For more detailed numbers see Table 2. aSAH, aneurysmal subarachnoid hemorrhage; IGF-I, insulin like growth factor-I.

Serum IGF-I concentrations are presented in Figure 1. IGF-I levels were significantly lower in patients with aSAH than in control patients on days one to five (*P *= 0.01), but no difference was found at three months follow up (Table 2). The mean IGF-I concentration for days one to five was 8.1 ± 3.5 nmol/l in patients with aSAH and 11.2 ± 3.1 in the control group. Serum GH concentrations were similar in patients with aSAH and control patients. Serum GH and IGF-I did not correlate at any time point in patients with aSAH or in the control group.

**Table 2:** GH and IGF-I concentrations at different time points

GH (mU/l)				IGF-I (nmol/l)		
	aSAH	Control	*P *value	aSAH	Control	*P *value
day1	3.4 ± 5.5	1.6 ± 2.7	0.18	8.2 ± 3.1	10.5 ± 2.7	0.04
day2	3.5 ± 5.5	4.5 ± 5.3	0.48	8.4 ± 3.6	11.9 ± 3.1	<0.01
day3	3.0 ± 5.3	1.8 ± 1.6	0.37	8.1 ± 4.2	11.4 ± 4.2	<0.01
day4	2.4 ± 4	3.5 ± 4.5	0.54	7.8 ± 3.9	10.9 ± 3.8	0.01
day5	2.0 ± 2.1	2.4 ± 3.7	0.72	8.0 ± 4.4	11.1 ± 4.1	<0.01
day6	2.1 ± 2.4			7.8 ± 4.0		
day7	1.6 ± 2.4			7.7 ± 4.0		
3 months	2.0 ± 5.1	3.7 ± 5.0	0.23	9.7 ± 3.1	10.4 ± 2.4	0.9

Data are presented as mean values ± standard deviations.

aSAH, aneurysmal subarachnoid hemorrhage;GH, growth hormone; IGF-I: Insulin like growth factor-I.

**Figure 2:**
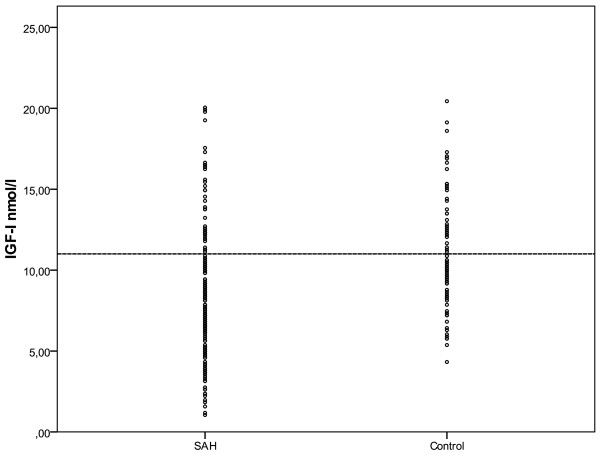
**Pooled IGF-I concentrations in patients with aSAH and in the control group**. The line represents the cut-off level of 11 nmol/l. The insulin like growth factor-I (IGF-I) concentration was below 11 nmol/l on day one in 77% of aneurysmal subarachnoid hemorrhage (aSAH) patients and in 23% (*P *= 0.02) of control patients. The respective values were 74% and 27% (*P *= 0.05) on day two, 70% and 30% (*P *= 0.3) on day three, 72% and 28% (*P *= 0.22) on day four, and 74% and 27% (*P *= 0.05) on day five. In the aSAH group, 83% of patients on day six and 76% on day seven had IGF-concentrations below 11 nmol/l. At three months, no patient had IGF-concentrations lower than 11 nmol/l.

Figure 2 represents the pooled distribution of IGF-I in respect to different cut-off levels.

No statistically significant differences were found in the proportion of having lower than age-related IGF-I reference values between patients with aSAH or controls. Hunt and Hess grade, Fisher grade, and the presence of hydrocephalus or vasospasm were not associated with the level of IGF-I concentration, either less than 11 nmol/l or more than 11 nmol/l.

In patients with aSAH, there were no differences in IGF-I or GH concentrations between the patients with respect to aneurysm location (anterior communicating artery versus others), treatment modality (clip vs. coil), Hunt-Hess grades (I to III versus IV to V), Fisher grade (I to II versus III to IV), Glasgow Coma Scale (GCS) (less than 8 or more than 8), symptomatic vasospasm and/or need for norepinephrine (n = 9), or hydrocephalus on admission. Gender or body mass index did not affect IGF-I or GH concentrations. Age was negatively correlated with IGF-I (r = -0.31, *P *≤ 0.001).

The mean 15D HRQoL sum in patients with aSAH was 0.81 ± 0.16 and in control patients 0.86 ± 0.09 (*P *= 0.24). Quality of life was moderately low (<0.8) in six patients with aSAH and in five control patients (*P *= 0.75). In addition, three patients with aSAH and none of the control patients had poor quality of life (<0.6). The HRQoL dimensions of speech (*P *= 0.04) and eating (*P *= 0.03) were lower in patients with aSAH than in the control group; otherwise, no statistically significant differences were observed in the scores between the groups. Ten patients in the aSAH group had a GOS of four or less, and the others had a GOS of five. Patients with a GOS of four or less had lower quality of life than patients with a GOS of five (0.7 vs.0.88, *P *= 0.003). Additionally, patients with a GOS of four or less had lower mean IGF-I concentrations than patients with a GOS of five (8.3 ± 2.6 nmol/l vs. 12.7 ± 5.1 nmol/l, *P *= 0.02). Patients with Hunt & Hess grades IV to V had similar HRQoL than patients with Hunt & Hess grades I to III (0.73 ± 0.2 vs. 0.84 ± 0.13, *P *= 0.12). aSAH patients with low HRQoL (<0.8) had lower mean IGF-I concentrations than patients with good HRQoL (5.7 ± 2.1 nmol/l vs. 8.7 ± 3.4 nmol/l). Mean IGF-I for days one to seven was mildly associated with HRQoL by a Pearson's correlation coefficient of 0.36 (*P *= 0.08).

In the Bayesian model, use of statins prior to aSAH, hyponatremia, high maximal sequential organ specific score, and the sum of IGF results for days one to seven after the aSAH were associated with poor HRQoL. This model was accurate in 78.6% of cases in leave-one-out cross validation (corresponding log-score 0.57, compared with 53.6% accuracy for the biggest class (i.e., default or educated guess), log-score 0.69). When the model was tested by putting the total material into the model, an accuracy of 89% was reached. The sensitivity was 0.86 and specificity was 0.93. The results indicate that a low IGF level in the days following aSAH may be associated with poor HRQoL or death.

## Discussion

To the best of our knowledge, this is the first study to evaluate the behavior of IGF-I in the acute phase of aSAH, and to combine it with GOS and HRQoL assessment at three months. According to our study, serum IGF-I levels are low during acute aSAH, but they normalize at three months. The results may reflect diminished acute pituitary function. The severity of aSAH does not influence serum IGF-I levels. Quality of life at three months was equal in patients with acute alive aSAH at three months and patients who underwent elective surgery for unruptured aneurysms. However, low IGF-I values, measured acutely after aSAH, may predict morbidity assessed by GOS and HRQoL.

aSAH may cause various long-lasting neurological deficits and disturbances in mental health, sleep, concentration capability, anxiety, and depression [24]. These symptoms are thought to be caused by aSAH-related ischemic lesions. There is some evidence showing pituitary function deficits may affect quality of life years after aSAH [9]. A GH deficiency might adversely affect vitality, sleep, and fatigue dimensions of quality of life. Although the mean quality of life was similar in patients with aSAH and control patients, patients with aSAH had depressed quality of life in eating and speech. Those aSAH patients with cumulative low IGF-I concentrations are at risk for low quality of life and increased morbidity, which may support the theory of the imperative role of IGF-I in the acute phase of neurological brain injury [15]. The role of IGF-I in the recovery of aSAH is not well studied; however, it is known that aSAH causes various long-lasting deficits in quality of life [24]. In our study, we found an association between IGF-I and HRQoL and hypothesize, but cannot demonstrate, that this is causal.

In the context of aSAH, it is most interesting that IGF-I presents with major vascular effects, and thus may contribute to the pathophysiological processes in aSAH [25].

Serum IGF-I is essential in mediating GH action. During critical illness, serum levels of IGF-I may decrease substantially [14]. There is increasing evidence that IGF-I has an essential role in neuronal growth, regeneration, and apoptotic cell death, and in adaptation to brain ischemia [10-12,15]. All these mechanisms may also be involved in acute aSAH [13]. In ischemic stroke, as well as meningococcal sepsis, low IGF-I concentrations may predict poor outcome in humans [15,26]. Surgical stress and brain manipulation in the control group were not associated with a decrease in IGF-I concentrations, as in patients with aSAH, regardless of the severity. Several factors other than GH secretion, for example nutritional status, may affect IGF-I concentrations [27]. Therefore, low IGF-I is not always associated with GH deficiency *per se *[28].

Low IGF-I concentrations in aSAH may reflect either pituitary GH insufficiency, decreased liver production, or a stress-related decrease of IGF-I concentrations in critical illness. An essential percentage of aSAH patients had IGF concentrations far below the age-related reference values. It was shown previously that a disturbance in the function of the GH-IGF axis is the first pituitary deficiency to appear after aSAH or TBI [4,29]. The mechanisms are not fully understood, but the vulnerable and complex vasculature of the pituitary gland may easily be affected by severe brain insults.

The role of single IGF-I concentration measurement may be controversial. It is generally accepted that definitive GH insufficiency should be tested by means of a stimulation test [30]. Concentrations of IGF-I less than 10 nmol/l have a specificity of 95%, but a sensitivity of only 40% in diagnosing GH deficiency [31]. In our study, the IGF-I concentrations were often far lower than this threshold. There are numerous cut-off points for IGF-I in screening for GH deficiency [19,28,32]. However, it is not known how high concentrations of IGF-I could be neuroprotective in acute intracranial neurological catastrophes.

In our study, GH concentrations and IGF-I concentrations did not correlate with each other. GH concentrations vary a lot during critical illness; the pulsatile secretion of GH makes adequate interpretation of serum concentrations of GH especially difficult. In the present study, GH concentrations were equal in patients with aSAH and the control patients. In the literature, there are no data on GH concentrations in the acute phase of aSAH. At the later phase, after aSAH, GH insufficiency may appear in 25% of patients [4].

There are some limitations in our study. We did not use the GH stimulation test when diagnosing GH deficiency. However, as described above, IGF-I itself has an essential role in acute neurological diseases [11,12]. The method used for measuring IGF-I may also influence the results [27]. Although our sample size was small, the aSAH was well defined by the onset and type of bleeding. The control group may be criticized because it consisted of patients undergoing open cranial surgery; that is, patients exposed to similar kinds of stress as those with aSAH. However, using other ICU patients as the control group might have caused other confounding factors, such as infections and hemodynamic instability.

We propose that it might also be argued that the method of securing the ruptured aneurysm in the aSAH group could markedly effect the results. However, severity of the bleeding and the level of consciousness on admission are by far the most important prognostic factors in aSAH and the treatment modality is of lesser importance [33,34]. As expected, there were no differences in IGF-I or GH concentrations between the patients with respect to treatment modality. If surgery had an effect, the surgical patients in the control would compensate this effect on IGF-I in comparisons between the control and aSAH groups.

IGF-I may be one factor among multiple others in influencing outcome in patients with aSAH. Larger studies are needed to prove the causality of this finding.

## Conclusions

Serum IGF-I concentrations are low in patients with acute aSAH, which may affect morbidity in these patients. The severity of aSAH did not affect IGF-I concentrations. Larger studies are needed to evaluate the neuroprotective role of IGF-I in aSAH.

## Key messages

• IGF-I has an important role in neuronal growth and cell death

• Serum IGF-I concentrations are low in the acute phase of aSAH

• Low IGF-I concentrations may reflect acute pituitary insufficiency in patients with aSAH

• Low IGF-I concentrations may affect morbidity in patients with aSAH

## Abbreviations

aSAH: aneurysmal subarachnoid hemorrhage; ELISA: enzyme-linked immunosorbent assay; GH: growth hormone; GCS: Glasgow Coma Scale; GOS: Glasgow outcome scale; HDU: high-dependency unit; HPA: hypothalamo-pituitary-adrenal; HRQoL: health-related quality of life; IGF-I: insulin-like growth factor-I; LOS: length of stay; TBI: traumatic brain injury.

## Competing interests

The authors declare that they have no competing interests.

## Authors' contributions

SB, AU and TK participated in study conception, acquisition of data, analysis and interpretation of data, drafting the manuscript and revising it critically. OR participated in analysis and interpretation of data, drafting the manuscript and revising it critically. ER, JRO and VK participated in analysis and interpretation of data.
